# Triazolam-induced Mania in a Patient with Bipolar I Disorder Following a Dental Procedure

**DOI:** 10.7759/cureus.7119

**Published:** 2020-02-27

**Authors:** Jasbir Singh, Brianna Skrzypcak, Rama Yasaei, Nishan Mangat

**Affiliations:** 1 Department of Psychiatry, University of California, Los Angeles-Kern Medical Center, Bakersfield, USA

**Keywords:** triazolam, mania, dental procedures, anxiety

## Abstract

Dental appointments and procedures may induce anxiety in some patients, which may ultimately lead to nonadherence and detrimental long-term effects. Often times, dentists prescribe short-acting benzodiazepines (triazolam) to alleviate anxiety during the duration of the procedure. However, benzodiazepines can cause adverse effects such as delirium and psychosis, which can be exacerbated by their interaction with previously prescribed medications and in those with mental health conditions.

Our patient, a 60-year-old Caucasian female, with stable bipolar I disorder presented to the inpatient psychiatric unit with psychotic features and was diagnosed with substance-induced mania secondary to the administration of triazolam by her dentist for upcoming procedures. The patient’s symptomatology resolved upon stabilization, and she was transferred to outpatient psychiatry for continued management.

## Introduction

Many patients report anxiety related to dental encounters, which may result in nonadherence to necessary appointments and procedures. Dentists may prescribe a short course of sedative and/or anxiolytic medications, commonly short-acting benzodiazepines (triazolam), to patients who have difficulty following through with dental procedures secondary to underlying anxiety [1]. Benzodiazepines enhance the effect of gamma-aminobutyric acid (GABA), an inhibitory neurotransmitter, by binding to the GABA-A receptor and increasing the frequency of chloride channel opening, thus facilitating its anxiolytic and sedative properties. These medications are metabolized by the cytochrome P450 enzymes to facilitate elimination [2]. Common side effects of benzodiazepines include sedation, anterograde amnesia, central nervous system (CNS) depression, and an increased risk of sleep-related activities [3]. Although not as common, respiratory depression, hallucinations, and delirium have been reported.

The adverse effects of benzodiazepines may be exacerbated by drug-drug interactions. For example, there is an increased risk of CNS side effects with the concomitant administration of opioids and cytochrome P450 inhibitor medications with benzodiazepines [2,4]. Therefore, it is important for healthcare professionals to obtain a thorough history regarding past medical and psychiatric conditions and current medications prior to prescribing benzodiazepines, especially when utilizing their anxiolytic proprieties for dental procedures. By obtaining this information, dentists may decrease the risk of adverse effects associated with the interaction of benzodiazepines with other medications. Literature has discussed the adverse effects of triazolam administered for dental procedures, and caution is warranted with concomitant medication use. In a case report published in 2005, a 49-year-old male experienced delirium and endorsed auditory and visual hallucinations following the administration of triazolam for anxiety related to a dental procedure [5]. The patient was prescribed triazolam 0.25 mg x 2. The patient’s psychotic symptoms were refractory to flumazenil, which is the antidote commonly used for benzodiazepine overdose. It was later determined that the patient was prescribed oxycodone and antibiotics, which may have interacted with triazolam and provoked the adverse effects. In addition, benzodiazepines may precipitate psychotic symptoms in those who have underlying psychiatric disorders, such as bipolar I disorder as seen in our patient. 

Bipolar I disorder is defined by the presence of at least one manic episode (mania), which lasts at least one week unless hospitalized. Mania is characterized by the presence of elevated or irritable mood along with increased energy, distractibility, impulsivity, grandiosity, flight of ideas and pressured speech, increased activity and psychomotor agitation, and decreased need for sleep. There is also marked functional social and occupational impairment from baseline. Mania commonly presents with psychotic features, including hallucinations or delusions, which are often paranoid and grandiose in nature. The management of bipolar I disorder involves the utilization of atypical antipsychotics (e.g., ziprasidone) for acute mania and mood stabilizers (e.g., lamotrigine, gabapentin) for maintenance [6]. Caution is warranted with the use of antidepressants and possibly benzodiazepines because they may precipitate mania. In our case report, we will investigate the explanations of triazolam-induced mania with psychotic features in a patient with bipolar I disorder and highlight the importance of obtaining a thorough medical history prior to prescribing benzodiazepines.

## Case presentation

Our patient is a 60-year-old Caucasian female with a past medical history of bipolar I disorder, insomnia, anxiety, and chronic neuropathic pain. The patient was prescribed ziprasidone 40 mg PO qAM and 80 mg qHS, lamotrigine 200 mg PO daily, mirtazapine 45 mg PO daily, cyproheptadine 4 mg PO daily, and gabapentin 400 mg PO TID. With this medication regimen, the patient’s bipolar I disorder was stable and controlled with her last manic episode being in 2003.

The patient reported a history of anxiety during dental procedures. As a result, her dentist prescribed triazolam 0.50 mg PO x 2 for two upcoming dental procedures that were scheduled two weeks apart and instructed the patient to take triazolam 0.50 mg PO 30 minutes before each procedure. For her first dental procedure, the patient took triazolam 0.50 mg PO as instructed. She reported decreased anxiety during the procedure and increased sedation and anterograde amnesia following the procedure. She returned back to baseline in approximately two days. The patient thought she took “too much of the medication” and decided to lower the dose of triazolam to 0.25 mg PO for her next scheduled dental procedure without notifying her dentist about the side effects. In preparation for her second dental procedure, the patient took triazolam 0.25 mg PO but missed her appointment secondary to experiencing manic symptoms (racing thoughts, insomnia, increased energy, delusions of reference). The patient experienced manic symptoms over the next four days and reported sleeping a total of four hours. The patient’s niece repeatedly called the patient for two days, decided to check on the patient after she was unable to get in contact with her, found the patient in altered mental status, and took the patient to a nearby clinic for evaluation. 

The patient was transferred to the inpatient psychiatric unit (IPU) and diagnosed with substance-induced mania with psychotic features secondary to triazolam. The patient was stabilized and placed back on her home mood stabilizer medications. The Clinical Institute Withdrawal Assessment (CIWA) protocol was followed for seizure prophylaxis secondary to benzodiazepine withdrawal. Over the next few days, the patient’s psychotic symptoms resolved, and she returned back to baseline. The patient was educated on the increased risk of adverse effects associated with short-acting benzodiazepines due to her prescribed medications and diagnosis of bipolar I disorder. The patient agreed to follow up with outpatient psychiatry upon discharge.

## Discussion

Our patient is a 60-year-old Caucasian female with a history of bipolar I disorder who presented with substance-induced mania with psychotic features secondary to triazolam administration by her dentist. There are multiple hypotheses to explain this adverse reaction: medication regimen and drug-drug interactions, prescribed dose of triazolam, age of 60 years, and diagnosis of bipolar I disorder.

The patient’s medication regimen along with the concomitant administration of triazolam may have precipitated mania. All of these medications in isolation interact with triazolam and are associated with an increased risk of CNS depression and/or psychomotor impairment [3]. For example, the dose of mirtazapine and/or triazolam should be decreased if they are administered together secondary to their additive effects. Our patient’s medication interactions with triazolam may be a possible explanation as to why she experienced mania with psychotic features; this could be an atypical presentation of CNS depression because there are not any drug-drug interactions indicating an increased risk of psychosis as an adverse outcome. When taking medication interactions into consideration, our patient may have benefited from the anxiolytic properties of gabapentin instead of triazolam as gabapentin does not exert amnesia and other significant CNS side effects after concomitant medication use [7].

It is suggested that “the usual recommended adult dose [of triazolam] is 0.25 mg” and should not exceed 0.50 mg secondary to adverse reactions. “In geriatric and/or debilitated patients the recommended dosage range is 0.125 mg to 0.25 mg [8].” The adverse effects may be dose-dependent and include sedation, abnormal thinking, behavior changes, decreased inhibition (excessive aggression and extroversion), bizarre behavior, agitation, and hallucinations [3]. Our patient was prescribed the maximum dose of triazolam (0.50 mg) and experienced manic symptoms: racing thoughts, insomnia, increased energy, and delusions of reference. The dentist should have considered prescribing a lower dose given her age, and subsequently, our patient may have been less susceptible to experiencing the adverse effects listed above.

The Beers Criteria for potentially inappropriate medication use in older adults was developed by the American Geriatric Society (AGS) to provide guidelines for healthcare professionals, including physicians and dentists, regarding the prescription of medications in older adults. According to the AGS, benzodiazepines should be used with caution in patients who are 65 years and older (independent of diagnosis or condition) due to an “increase[d] risk of cognitive impairment, delirium, falls, fractures, and motor vehicle [accidents] [9].” In the elderly, these risks are increased secondary to decreased rates of metabolism [3]. Although our patient does not fit the criteria of being elderly (age 65 years and older), she is close in age and is at increased risk of these adverse effects, which was seen following the administration of triazolam. Thus, a different medication should have been considered in our patient and in elderly patients who experience anxiety surrounding dental procedures. 

In psychiatric patients, benzodiazepines can lead to undesirable outcomes, such as substance-induced mania with psychotic features as seen in our patient who had a diagnosis of stable bipolar I disorder prior to the administration of triazolam. A double-blind clinical study was conducted over four weeks to study the side effect profile of benzodiazepines in five psychiatric inpatients [10]. Patients were administered triazolam for two weeks, which was preceded by a placebo for one week and followed by a placebo for one week to determine baseline. During the period of triazolam administration, “psychopathology greatly intensified across all of the patients with the emergence of anxiety, memory impairment, confusion, paranoid ideation, and hallucinations,” which gradually subsided [10]. This study demonstrates that benzodiazepines in isolation can contribute to adverse effects seen in patients with known psychiatric conditions. This highlights the importance of gathering a comprehensive medical history, including psychiatric conditions, prior to prescribing benzodiazepines for dental procedures.

Evidence suggests the increased risk of CNS depression and psychomotor impairments with the concomitant administration of benzodiazepines with other medications, but there is not any literature supporting the adverse effect of mania as seen in our patient. Future research is indicated to study the effect that benzodiazepines, in isolation or in combination with other medications, have on inducing mania in patients with and/or without psychiatric conditions.

## Conclusions

Our patient, a 60-year-old female, with stable bipolar I disorder presented to the IPU with psychotic features and was diagnosed with substance-induced mania secondary to the administration of the maximum recommended dose of triazolam by her dentist to alleviate anxiety for upcoming procedures. Our patient's symptomatology may have been an atypical presentation of CNS depression and psychomotor impairment, which are adverse effects of benzodiazepines. This case report demonstrates the importance of gathering a complete history and investigating drug-drug interactions prior to prescribing benzodiazepines to patients, which may decrease the risk of associated adverse effects. 
